# The Long-term Radiographic Fate of the Chronically ACL-Deficient Knee: A Systematic Review and Meta-analysis of Matched Cohort Studies

**DOI:** 10.1177/03635465251405438

**Published:** 2026-01-23

**Authors:** Ajay Shah, Kosaran Gumarathas, Paul Marks, Robert G. Marx, Alexander Kiss, David Wasserstein

**Affiliations:** † Division of Orthopaedic Surgery, Faculty of Medicine, University of Toronto, Toronto, Canada; ‡ Division of Orthopaedic Surgery, Sunnybrook Health Sciences Centre, University of Toronto, Toronto, Canada; § Sports Medicine Institute, Department of Orthopaedic Surgery, Hospital for Special Surgery, New York, New York, USA; ‖ Holland Bone and Joint Program, Sunnybrook Research Institute, Sunnybrook Health Sciences Centre, Toronto, Canada; Investigation performed at University of Toronto, Toronto, Canada

**Keywords:** anterior cruciate ligament, knee osteoarthritis, nonoperative management, ACL reconstruction

## Abstract

**Background:**

Determining the long-term risk of arthritis in patients with anterior cruciate ligament (ACL) injury treated nonoperatively versus those who undergo ACL reconstruction (ACLR) remains an important and unanswered question for patients and surgeons.

**Purpose:**

(1) To define the cumulative arthritis rate and severity after nonsurgical management of ACL injury—the chronically ACL-deficient (ACLD) knee; (2) to compare rates and severity of arthritis in patients who have ACLD knee with similar patients who underwent ACLR; and (3) to identify clinically relevant risk factors for arthritis.

**Study Design:**

Systematic review; Level of evidence, 3.

**Methods:**

Three databases (Medline, Embase, PubMed) were searched for primary studies examining radiographic outcomes in patients with chronic ACL deficiency (>12 months of ACL deficiency). Studies with a matched ACLR control group were included. Quality assessment was performed with the MINORS (Methodological Index for Nonrandomized Studies) tool. Arthritis prevalence over time was plotted and modeled to best-fit using the Akaike information criterion. Data were extracted for meta-analysis for the primary outcome of osteoarthritis. The cumulative odds ratio of prognostic factors was calculated where appropriate.

**Results:**

Nineteen full-text studies met inclusion criteria (11 matched cohort studies comparing ACLD and ACLR) including 1432 patients with a mean 11.1 years of follow-up after injury. The methodological quality of included studies was moderate. The pooled rate of radiographic arthritis in ACLD patients was 37.8%; the rate of moderate to severe arthritis was 18.1% (compared with 35.2% and 12.8% in patients with ACLR, respectively, and 5.0% in the nonoperated knee). An increase in the rate of arthritis was observed, accelerating sharply at 10 years after injury. ACLR and ACLD knees had similar prevalence of mild arthritis (*P* = .60), irrespective of activity level. Joint degeneration was significantly accelerated by meniscectomy in ACLD patients in most studies.

**Conclusion:**

Patients with a chronically ACLD knee may be at an increased predisposition for developing moderate to severe arthritis but not mild arthritis compared with matched patients who undergo ACLR. Meniscectomy is a key predictor of worsened severity of osteoarthritis.

The anterior cruciate ligament (ACL) is the primary restraint to anterior tibial translation and internal tibial rotation at the knee.^5^ ACL rupture is the most common complete ligament injury of the knee.^10^ The annual ACL injury incidence is estimated to be as high as 85 in 100,000 people aged 16 to 39 years.^24^ Surgical ACL reconstruction (ACLR) is often performed in patients with the goal of restoring sagittal and rotational stability.^19^ Preclinical studies show that ACL transection leads to increased tibial anterior translation and internal rotation, causing anterior subluxation of the lateral tibiofemoral compartment, clinically resulting in patients experiencing the pivot-shift phenomenon.^35,51^

However, many patients with ACL rupture do not undergo surgery, for reasons including personal preference, activity level, symptoms, or surgeon recommendation.^2,9^ This patient could be older and lower demand, could participate in noncutting sports, or could simply be classified as a “coper” or “adaptor.”^36^ These patients are usually managed with staged physiotherapy and lifestyle modifications to achieve functional stability and return to activity. Several landmark prospective studies (eg, KANON, Delaware-Oslo) have purposefully compared early ACLR versus rehabilitation with possible late ACLR and reported that many patients can achieve good knee function without surgery.^13,17^ However, the long-term fate of a nonoperatively treated ACL-deficient (ACLD) knee is less well understood.

The pathophysiological process of posttraumatic knee osteoarthritis (OA) is complex. OA is reported after both operative and nonoperative management of ACL injuries.^9^ OA is diagnosed using plain radiographs and graded using validated scales, such as the Kellgren-Lawrence (KL) classification. A common question from a patient with acceptable knee function after ACL injury is “Will ACLR prevent the development of arthritis in my knee?” Previous cohort studies have shown radiographic OA rates of 60% to 90% at 10 to 15 years in nonoperatively treated patients,^4,15,33,45^ whereas 10-year data from the MOON cohort reported OA rates of 23% to 37% at 10 years after ACLR.^31^ However, in 2 recent systematic reviews, Cuzzolin et al^7^ found no difference in OA at 5 to 10 years after injury between ACLR and nonoperative patients, whereas de Jonge et al^8^ found lower rates of OA in nonoperatively treated knees (odds ratio [OR], 1.84). Limitations of these systematic reviews included small sample sizes (n = 5 and 8 studies, respectively) and high risk of bias.

The purpose of the current study was to systematically review the literature pertaining to radiographic arthritis observed in patients with a chronic ACLD knee, calculate the cumulative rate of OA in the ACLD knee, compare it with a matched cohort of ACLR knees using meta-analysis, and identify risk factors for the development of arthritis.

## Methods

### Search Strategy

Three online databases (Embase, PubMed, and Ovid [Medline]) were searched for literature from database inception until October 31, 2024, for studies pertaining to radiographic OA in the ACLD knee. The search included the following terms: anterior cruciate ligament, x-ray, radiograph, and chronic (Appendix Table A, available in the online version of this article). References of included studies were hand-searched using a snowball screening method, and authors of published conference abstracts were contacted to request data sets.

### Study Screening

The titles, abstracts, and full-text articles were screened by 2 reviewers (A.S. and K.G.) independently and in duplicate. Disagreements during title and abstract screening moved on to the next stage for more in-depth review. Any disagreements were discussed between reviewers, and a senior author (D.W.) was consulted for any remaining discrepancies. The references of the included studies were subsequently manually screened for additional articles that may have eluded the initial search strategy.

### Assessment of Study Eligibility

The research question and study eligibility criteria were established a priori. The inclusion criteria were studies investigating humans with ACLD knee, studies with level of evidence 1 to 3, and studies examining radiographic arthritis. ACLD knee was defined as a knee with ACL rupture that was treated without reconstruction, with minimum follow-up >12 months from the time of injury. Cohort studies with ACLD and ACLR subgroups were included. Exclusion criteria were animal studies, commentaries, book chapters, review articles, and technical studies.

### Data Abstraction

Data were collected by 2 reviewers and recorded in a Microsoft Excel spreadsheet (Version 2007; Microsoft). Abstracted data included authors, year of publication, study design, sample sizes, sex ratio, mean age, ACLD and comparison group demographic characteristics, follow-up time, radiographic outcomes (severity and prevalence of OA), key findings, and conclusions.

### Quality and Agreement Assessment

The methodological quality of the included studies was assessed in duplicate using the MINORS (Methodological Index for Nonrandomized Studies) instrument. This tool was designed to assess the methodological quality of comparative and noncomparative, nonrandomized studies. For noncomparative studies, an ideal score was 16 points; for comparative studies, the maximum score was 24 points. For noncomparative studies, a MINORS score of <8 was considered poor quality, 9 to 14 moderate quality, and 15 to 16 good quality.^40^ For comparative studies, a score of <14 was considered poor quality, 15 to 22 moderate quality, and 23 to 24 good quality.^18^

To assess the interreviewer agreement, a kappa (κ) statistic was calculated for the title, abstract, and full-text screening stages. Agreement was categorized a priori as follows: κ/intraclass correlation coefficient (ICC) ≥0.61 was considered substantial agreement; κ/ICC 0.21 to 0.60, moderate agreement; and κ/ICC ≤0.20, slight agreement.^2^

### Statistical Analysis

Information regarding the prevalence and severity of arthritis in each study was extracted and plotted alongside with the study follow-up period. Using the Akaike information criterion,^3^ the error (*R*^2^) from 5 models (linear, quadratic, cubic, logarithmic, and exponential) was calculated, and the model with the smallest error was selected and graphed. When sufficient comparative cohort data were available for pooled meta-analysis, the analysis was performed using the RevMan software (Cochrane Collaboration) using random-effects models and a significance of .05. Descriptive statistics including mean, proportion, standard deviation, and 95% CI were calculated using Minitab statistical software (Version 17).

## Results

### Search Strategy

From 2187 initial studies, 19 full-text articles met inclusion and exclusion criteria (Appendix Figure A1, available online). The reviewers reached substantial agreement at the title and abstract (κ = 0.81; 95% CI, 0.70-0.92) and full-text (κ =1.00) screening stages.

### Study Quality and Characteristics

We identified 11 comparative studies^¶^ and 8 longitudinal cohort studies,^#^ all of which were levels 1 to 3 evidence (Appendix Table B1, available online). The mean MINORS score was 9.8 of 16 (range, 8-12) for the noncomparative studies and 19.2 of 24 (range, 14-23) for the comparative studies, suggesting moderate quality of evidence. A total of 1432 patients were included (20.3% female; n = 302).

Mean ± SD age at follow-up was 28.5 ± 6.9 years. In total, 813 knees were ACLD, and 619 were controls (ACLR n = 395, contralateral healthy knee n = 184, other n = 40). Mean follow-up time (from ACL injury to assessment) was 11.1 ± 6.8 years (range, 5-24.1 years). All ACLR studies (n = 11) reported standard ACLR with mostly bone–patellar tendon–bone grafts; ACLR technique details are found in Appendix Table C (available online).

Study follow-up periods were 4 to 7 years (n = 6), 7.1 to 10 years (n = 3), 10.1 to 15 years (n = 4), 15.1 to 20 years (n = 3), and >20 years (n = 3) (Table 1; Appendix Table B, available online). The mean Tegner score in this sample was 7.0 (n = 219; range, 5-9), corresponding to a recreational athlete.

**Table 1:** Summary of Study Findings*^*a*^*

Lead Author (Year)	Summary
Prospective Comparative Studies	
Fink^12^ (2001)	In chronic ACLD managed nonoperatively, patients developed similar mild to moderate degenerative changes compared with the operative group; however, a significant correlation was seen between participation in high-risk pivoting sports and osteoarthritic changes (*r* = 0.64) in the nonoperative group only, as well as a correlation between the degree of arthrosis and amount of meniscal resection performed during initial arthroscopy.
Frobell^13^ (2013)	At 5 years, no difference was seen in radiographic arthritis between ACLD, early ACLR, and delayed ACLR groups: 20 (51%) patients initially in a nonoperative pathway had eventual delayed ACLR.
Kessler^22^ (2008)	After 11 years of nonoperative management for isolated ACL rupture, 24% of patients developed clear OA (grade ≥II), with 61% showing no radiographic changes, 14% showing doubtful changes, and 24% demonstrating definite arthritic changes (20% grade II, 4% grade III); higher body mass index and age were significantly correlated with increased OA rates.
Meuffels^28^ (2009)	At a 10-year follow-up with high-level athletes with ACL injuries, radiographic OA (Kellgren-Lawrence grade ≥2) was found in 28% of nonoperatively treated knees compared with 48% of reconstructed knees, although this difference was not statistically significant (*P* = .145). In contrast, only 4% of uninjured contralateral knees showed radiographic changes.
Mihelic^30^ (2011)	At 17-20 years of follow-up, chronically ACLD knees managed nonoperatively showed significantly worse outcomes with 56% developing severe OA and 84% having abnormal/severe laxity, particularly in cases with concurrent meniscal injuries.
Myklebust^32^ (2003)	In chronic ACLD managed nonoperatively, radiographic assessment at 6-11 years showed gonarthrosis in 46% of cases, with similar rates to surgically treated patients (42%), although notably there was no correlation between radiographic findings and clinical pain scores.
Neuman^34^ (2008)	In chronic ACLD managed nonoperatively for 15 years, radiographic tibiofemoral OA developed in 16% of patients, with all cases occurring in those who underwent meniscectomy. Importantly, patients without meniscectomy showed no radiographic OA, suggesting meniscal preservation may be crucial for preventing OA in chronic ACLD.
Pedersen^38^ (2021)	In patients who chose nonoperative management with rehabilitation alone (65/262, or 25% of cohort), only 1/54 (2%) showed radiographic knee OA (Kellgren-Lawrence grade ≥2) at 5 years, which was not significantly different from surgical groups (8/113 [7%] for early ACLR, 3/20 [15%] for delayed ACLR), and similar findings were seen with joint space width measurements showing no significant differences between treatment approaches.
Streich^47^ (2011)	In chronic ACLD, in knees managed nonoperatively over 15 years, 55% developed progressive radiographic changes. The study showed that nonoperative treatment resulted in similar radiographic OA rates compared with reconstructed knees, with rotational instability (positive pivot shift) being the key factor associated with OA development rather than the treatment choice itself.
Tsoukas^48^ (2016)	After 10 years of chronic ACLD, no significant difference was seen in radiographic OA development between surgically and nonsurgically managed knees (23.5% vs 33.3%, respectively).
Van Yperen^50^ (2018)	After 20 years of follow-up, no significant radiographic differences were seen between athletes who chose operative vs nonoperative treatment for ACLD (*P* = .508).
Prospective Cohort Studies	
Fairclough^11^ (1990)	The earliest radiographic change in ACLD was the appearance of a small osteophyte in the intercondylar notch adjacent to the medial tibial spine. Progression to more obvious osteophyte formation on the spines and in the notch may occur. Repeated episodes of subluxation will further progress degeneration of the knee.
Gföller^14^ (2019)	In chronic ACLD managed nonoperatively over 20 years, patients developed significant arthritic changes (58.8%) compared with the contralateral knee, with the most pronounced changes in the medial compartment. Although sports activity level did not correlate with arthritis development, a significant correlation was found between initial lateral meniscal resection and subsequent lateral compartment arthritis.
Kannus^20^ (1989)	In chronic ACLD managed nonoperatively over 8 years, radiographic changes included osteophytes and subchondral sclerosis primarily affecting the medial compartment, with complete ACL tears showing 4-fold more severe changes compared with partial tears (70% vs 14% developed clear OA), suggesting that the amount of pathological osteoarthritic change depends on the degree of instability.
Konrads^23^ (2016)	In a select group of ACLD patients treated nonoperatively and followed for 27 years, radiographic arthritis (assessed by Sherman scores) significantly correlated with the presence of initial meniscal tears, worse Knee injury and Osteoarthritis Outcome Scores, and increased symptoms/pain. Despite this, 90% of patients rated their knee as normal or almost normal, with only 5% requiring secondary meniscal surgery during the follow-up period.
McDaniel^27^ (1983)	A definite increase was seen in roentgenographic changes of joint space narrowing and frank OA. It appeared that patients most at risk to develop these changes were overweight, had undergone a medial meniscectomy, and had a varus deformity of the injured knee.
Pattee^37^ (1989)	In chronic ACLD managed nonoperatively for 4-10 years, 65% of evaluated knees showed mild radiographic changes including femoral condyle flattening and early degenerative changes, whereas 35% remained radiographically normal. However, the limited number of patients with radiographic evaluation prevented definitive conclusions about long-term radiographic outcomes.
Sherman^42^ (1988)	Radiographic findings from chronic ACLD knees (nonoperative management) demonstrated progressive degenerative changes, with 72% (91/127 knees) developing medial tibial osteophytes and 26% (33/127 knees) showing joint space narrowing. The study suggested that degenerative changes worsen over time, with significant deterioration in radiographic scores after approximately 10 years of ACLD.
Shirakura^43^ (1995)	In chronic ACLD managed nonoperatively, radiographic changes were relatively mild, with only 17% (8/46) showing grade 1 osteoarthritic changes.

a ACL, anterior cruciate ligament; ACLD, anterior cruciate ligament deficiency; ACLR, anterior cruciate ligament reconstruction; OA, osteoarthritis.

### Pooled Arthritis Rate

Eleven matched-cohort studies compared ACLD knees to a matched ACLR control group. Common outcome measures included the KL system (n = 4), International Knee Documentation Committee (IKDC) classification (n = 3), and Fairbank classification (n = 2) (Appendix Tables D and E, available online). The definition of “arthritis” was considered KL ≥2, IKDC ≥B, whereas “moderate-to-severe arthritis” was KL ≥3, IKDC ≥C.

The pooled rate of “all arthritis” (KL ≥2; IKDC ≥B) in ACLD patients was 37.8% (225/596); moderate-to-severe arthritis was 18.1% (KL ≥3; IKDC ≥C; 75/415). The corresponding rates of all OA and moderate-to-severe OA in ACLR patients were 35.2% (195/553) and 12.8% (47/368), respectively. Longitudinal studies found that 5.0% (10/220) of contralateral uninjured knees had OA (ACLD group, odds radio 7.2).

**Figure 1.**
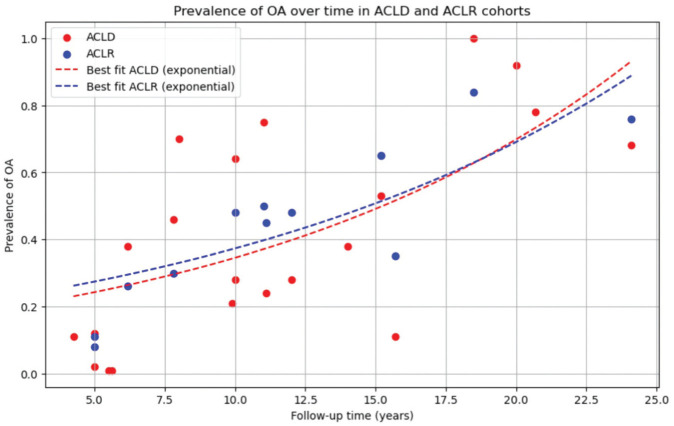
Prevalence of arthritis in ACLD patients over time. ACLD knee: *y* = 0.171 * exp(0.070 *x), *R*^2^ = 0.43. ACLD, anterior cruciate ligament–deficient; ACLR, anterior cruciate ligament reconstruction; OA, osteoarthritis.

**Figure 2.**
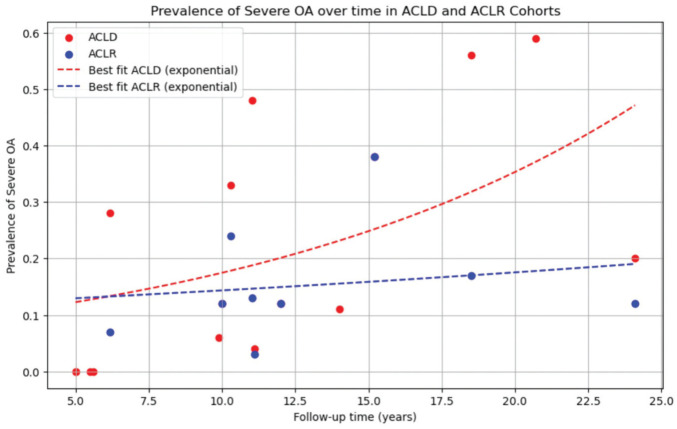
Prevalence of moderate-to-severe arthritis in ACLD patients over time. ACLD knee: *y* = 0.086 * exp(0.070 *x). ACLD, anterior cruciate ligament deficient; ACLR, anterior cruciate ligament reconstruction; OA, osteoarthritis.

Similar trends were observed between groups for “all arthritis” (Figure 1), but the ACLD group showed more rapid development of moderate-to-severe arthritis (Figure 2) over time.

Both graphs were best fit with an exponential line of best fit (*R*^2^ = 0.43), which estimated 50% prevalence for “all arthritis” at ~15.3 years after injury, and moderate-to-severe arthritis at ~25.2 years in ACLD knees (other models in Appendix Tables C and F, available online). An increase in the rate of arthritis was observed, accelerating sharply after 10 years postinjury.

### Meta-analysis

**Figure 3.**
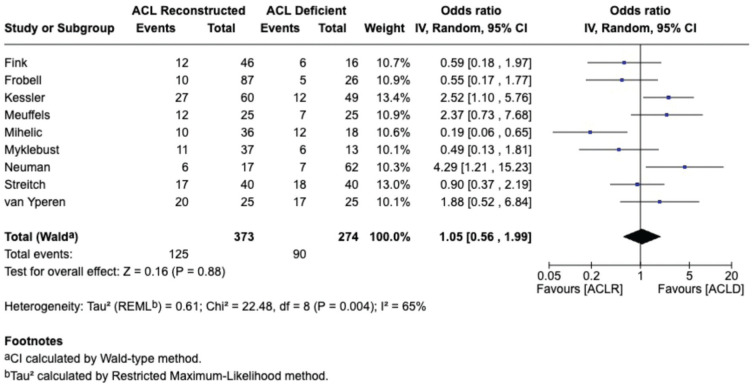
Meta-analysis of arthritis rates in anterior cruciate ligament–reconstructed (ACLR) vs anterior cruciate ligament–deficient (ACLD) knees.

**Figure 4.**
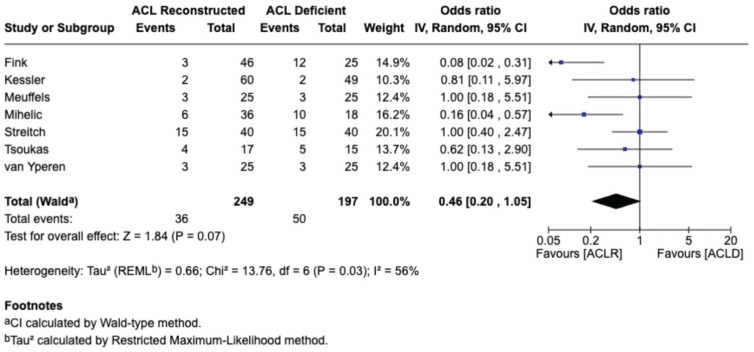
Meta-analysis of moderate-to-severe arthritis rates in anterior cruciate ligament–reconstructed (ACLR) vs anterior cruciate ligament–deficient (ACLD) knees.

Matched-cohort studies (equivalent baseline demographic characteristics: age, body mass index [BMI], sex, and activity level) of ACLD versus ACLR knees were compared using meta-analysis. For the development of “all arthritis” (KL ≥2; IKDC ≥B), surgical treatment (ACLR) had no preventive effect (n = 647; OR = 1.05 [range, 0.56-1.99]; *P* = .88) (Figure 3). Subanalyses of competitive (Tegner >7) and recreational (Tegner ≤7) athletes both showed no significant effect (Appendix Figures A2 and A3, available online) (*P* = .85 and *P* = .63, respectively). Meta-analysis of ACLD versus ACLR for moderate-to-severe arthritis (KL ≥3; IKDC ≥C) showed no statistically significant difference (n = 446; OR = 0.46 [range, 0.20-1.05]; *P* = .07) (Figure 4) of ACLR on arthritis development.

### Prognostic Factors

Meniscal injury and surgery were the most commonly reported positive prognostic factors for increasing the development of OA. Mihelic et al^30^ (17-20 years postinjury) reported higher rates of moderate-to-severe arthritis in ACLD knees with meniscal injury (44% vs 22%) versus ACL injury alone. However, surgical meniscectomy rates during the follow-up period did not differ between groups: ACLD knee (81/202; 40.1%), ACLR knee (97/264; 36.7%; *P* = .46).

Three comparative studies^12,34,50^ and 4 cohort studies^14,23,27,42^ reported a positive correlation between meniscectomy and arthritis; 2 studies reported no relationship.^37,47^ Meta-analysis of comparative studies was not possible due to incomplete outcome reporting.

No other factors were consistently predictive of arthritis. Considering other associated injuries, Neuman et al^34^ found that medial collateral ligament injury (grade 1-3) did not influence arthritis rates, and Sherman et al^42^ reported that the presence of a collateral ligament injury did not influence radiographic appearance.

Studies reporting some positive associations with arthritis include Fink et al^12^ (participation in pivoting sports; *r* = 0.64; *P* < .05); Kessler et al^22^ (age [>10 years: OR = 1.7] and BMI [OR = 1.2]); Streitch et al^47^ (positive pivot-shift); McDaniel and Dameron^27^ (higher BMI, age, restricted sport activity, worse patient-reported outcome measure [PROM] scores, and varus knees), and Sherman et al^42^ (longer time since injury, increased age, medial meniscectomy, and worse PROM scores). Studies reporting no significant associations include Streitch et al^47^ (KT-1000 arthrometer measurements, age, sex, BMI, time from injury, and PROM scores), Tsoukas et al^48^ (age), Myklebust et al^32^ (PROM scores: IKDC Pain, Lysholm), and Gföller et al^14^ (degree of sport participation).

## Discussion

The most significant finding in this systematic review is that patients with a chronic ACLD or ACLR knee had a higher rate of arthritis than their contralateral uninjured knee (37.8% and 35.2% vs 5.0%, respectively; OR = 7.6). ACLR did not show statistically significant differences to ACLD for the development of moderate-to-severe, or all, OA. Further, patients undergoing meniscectomy had consistently higher rates of arthritis (n = 8/10 studies). Radiographic changes were not consistently correlated with activity level (Tegner score), clinical instability, or PROMs in any study (Table 1).

The rate of arthritis reported in our study was comparable to other published studies in ACLD and ACLR cohorts. A large cohort study of all patients with ACL injuries reported a 40% rate of arthritis at 15 years and 90% at 30 years.^25^ In a systematic review of 9 studies in 2014, Ajuied et al^1^ reported that 31.5% of ACLD and ACLR patients had grade ≥2 KL changes on radiograph and 20.3% had KL ≥3 changes, compared with 4.9% in the control group at 10 years of follow-up. Their meta-analysis (6 studies) reported that “any arthritis” was more prevalent in ACLD knees (risk ratio [RR], 4.98) versus ACLR knees (RR, 3.62), but moderate-to-severe OA was more prevalent in ACLR knees (RR, 4.71) than ACLD knees (RR, 2.41). This contradictory finding was attributed to returning to sport, but the study was limited by a lack of data collection around activity levels and the inclusion of nonrepresentative samples. Our study contrasted these conclusions by showing no relationship between activity level and OA and including a longer duration of follow-up with more than twice as many studies and patients. The 10-year follow-up results of the Delaware-Oslo ACL cohort showed a lower rate of only 8% (KL ≥2) in the rehabilitation-only group, but this value increased to 35% when patients with any osteophytes were included.^49^ In our study, ACLD and ACLR groups had similar levels of mild arthritis (KL ≥2; IKDC ≥B), but the ACLD group had higher levels of moderate-to-severe arthritis (KL 3-4; IKDC C-D), suggesting that the degree of advanced joint degeneration may accelerate earlier in this group and that ACLR may be protective.

Several other studies found no difference in rates of arthritis between patients who had ACLR versus nonoperatively managed ACL tears.^1,8^ One study also found higher rates of meniscal tears in ACLD knees.^29^ Meniscal status plays an important role in modulating the development of moderate-to-severe arthritis. Without an ACL, the stabilizing influence of the menisci becomes even more important to knee kinematics, and the loss of this secondary stabilizer leads to a “double hit” of knee instability, altered biomechanics, and contact pressures. Grassi et al^16^ found that disruption to the medial meniscus predisposed the knee to greater anterior tibial translation, whereas lateral injuries affected rotational laxity. There are also reports of very high (>95%) rates of meniscal lesions and grade IV cartilage lesions (68%) in ACLD knees at 20 years.^33^ Intra-articular knee preservation of soft tissue structures is perhaps the strongest argument in favor of prompt ACLR, as delays >90 days or >12 months are consistently associated with higher rates of secondary meniscus and chondral lesions.^21,39,46^

It is therefore not surprising that meniscectomy was strongly predictive of arthritis in ACLD knees (8/10 studies). Although other studies of ACLR knees with meniscectomy showed higher rates of arthritis, they typically had a small effect size and shorter follow-up: Skinner et al^44^ reported a 1.45 OR of arthritis at 5 years postsurgery for ACL with meniscectomy.^6,26^ Unfortunately, our data lacked the sample size to conduct a meta-analysis of this outcome and to determine whether meniscectomy explained the difference in moderate-to-severe arthritis in ACLR and ACLD groups, and future large primary studies such as the Delaware-Oslo cohort may better answer this question.

Our initial clinical question was how to counsel patients who consider the risk of developing arthritis in their decision-making process to undergo ACLR. Patients wonder whether ACLR will prevent arthritis. Based on the data in this systematic review, the patient can be counselled as follows:

1. ACL injury is the strongest factor influencing the risk of developing arthritis, regardless of treatment. The pooled rate of OA was 37.8% in ACLD patients and 35.2% in ACLR patients, compared with 5.0% in contralateral uninjured knees.
2. Reconstruction and nonoperative care lead to similar rates of mild and moderate-to-severe arthritis at 11.1 years after injury regardless of activity level (eg, competitive vs recreational sport).
3. The status of a patient’s meniscus seems to have the greatest importance in the risk of developing OA in patients with complete ACL rupture, although more high-quality data are needed to draw pooled statistical conclusions.

A discussion surrounding patient goals and expectations, particularly around the option for delayed ACLR, should be conducted with every patient. Ten-year results from the Delaware Oslo cohort found that a delayed ACLR group had lower Knee injury and Osteoarthritis Outcome Scores and hop performance compared with ACLD and early ACLR groups but similar radiographic scores.^49^ However, the question of long-term function remains less well studied.

### Limitations

The primary limitation to the validity and generalizability of the conclusions of this study is that this is a secondary analysis of published data. This limits the specificity of data analysis, although it was controlled by including only matched-cohort studies in pooled analysis. We were unable to conduct certain important analyses, such as determining whether meniscal injury accounts for the difference between ACLD and ACLR in rates of moderate-to-severe arthritis. Another limitation is the relatively low reporting quality of included studies. Many (8/20) studies are >20 years old and have varied methodologic and reporting biases. For example, some used historical outcomes measures for radiographic arthritis (eg, Sherman score). Additionally, the quality of power calculations, outcome reporting, and statistical tests varied. There was heterogeneity of ACLR techniques, secondary procedures (in ACLD and ACLR patients), and nonsurgical rehabilitation protocol in ACLD. This may result in overestimation of OA rates, as modern anatomic ACLR is shown to have lower rates of OA in long-term follow-up.^41^

## Conclusion

Patients with a chronic ACLD knee had an increased predisposition for developing arthritis (mild, 37.8%; moderate-to-severe, 18.1% at 11.1 years) after injury compared with their uninjured knee and the general population. Patients with ACLR had no statistically significant difference in rates of mild and moderate-to-severe arthritis. Activity levels (Tegner score) did not affect arthritis rates, but meniscectomy was a strong predictor of worsened OA severity in most studies.

## Supplemental Material

sj-docx-1-ajs-10.1177_03635465251405438 – Supplemental material for The Long-term Radiographic Fate of the Chronically ACL-Deficient Knee: A Systematic Review and Meta-analysis of Matched Cohort Studies Supplemental material, sj-docx-1-ajs-10.1177_03635465251405438 for The Long-term Radiographic Fate of the Chronically ACL-Deficient Knee: A Systematic Review and Meta-analysis of Matched Cohort Studies by Ajay Shah, Kosaran Gumarathas, Paul Marks, Robert G. Marx, Alexander Kiss and David Wasserstein in The American Journal of Sports Medicine

## Footnotes

¶ References 12, 13, 22, 28, 30, 32, 34, 38, 47, 48, 50.

# References 11, 14, 20, 23, 27, 37, 42, 43.
