# Supplementary material for: The Long-term Radiographic Fate of the Chronically ACL-Deficient Knee: A Systematic Review and Meta-analysis of Matched Cohort Studies
Source: Am J Sports Med. 2026 Jan 23;54(8):2021–9. doi: 10.1177/03635465251405438 (PMC13279981; doi:10.1177/03635465251405438)
Supplement: sj-docx-1-ajs-10.1177_03635465251405438 – Supplemental material for The Long-term Radiographic Fate of the Chronically ACL-Deficient Knee: A Systematic Review and Meta-analysis of Matched Cohort Studies [file sj-docx-1-ajs-10.1177_03635465251405438.docx]

**Appendix Figures:**


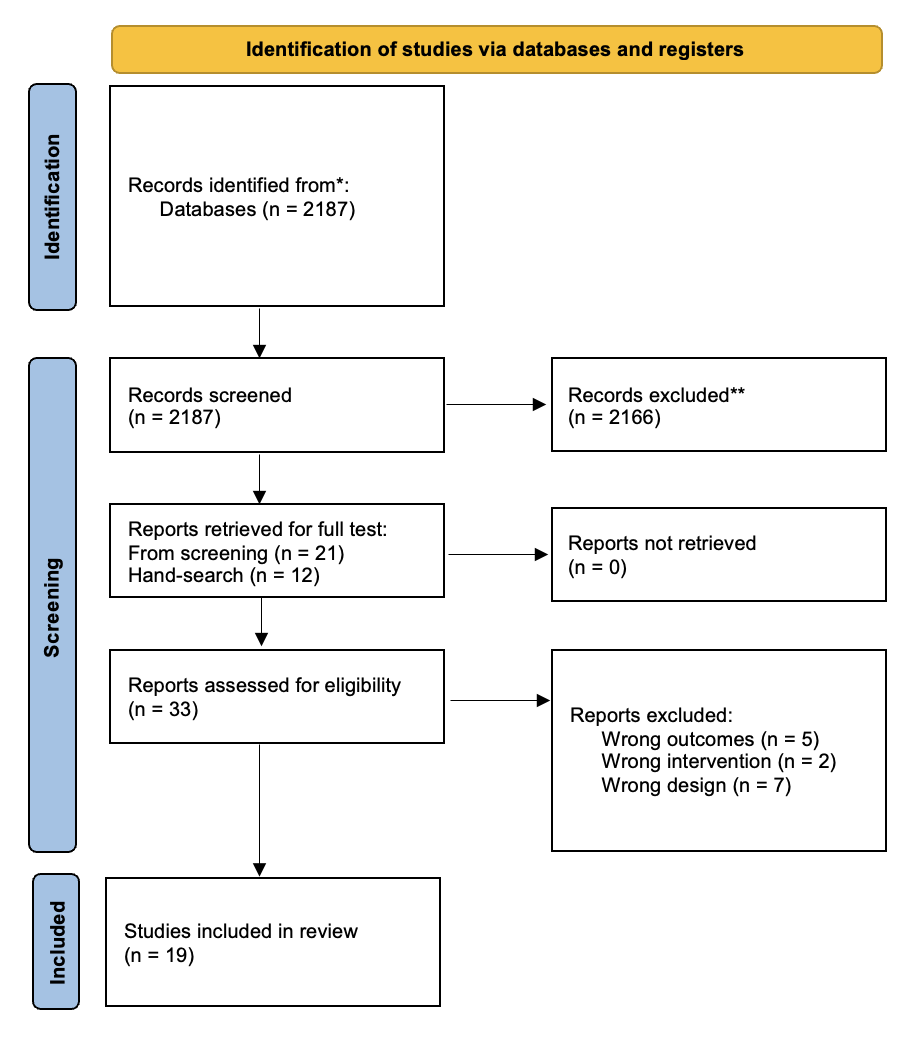


Appendix Figure 1. PRISMA Flow diagram demonstrating the systematic review of the literature for primary studies investigating chronically ACL deficient knees


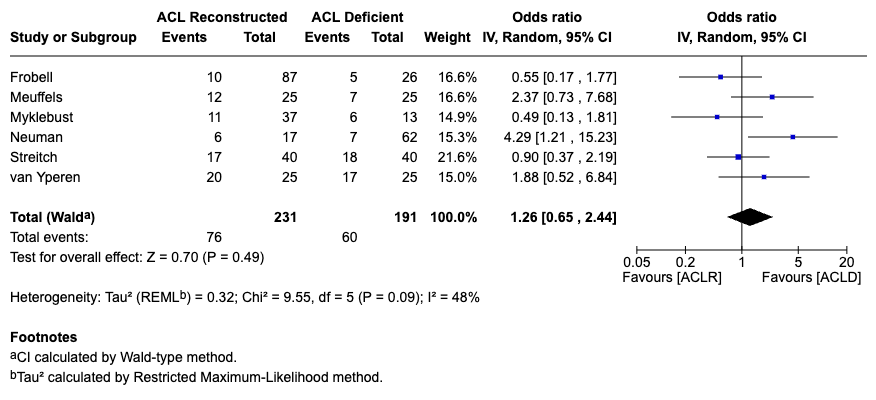


Appendix Figure 2. Meta-analysis of high-impact athletes (Tegner >7)


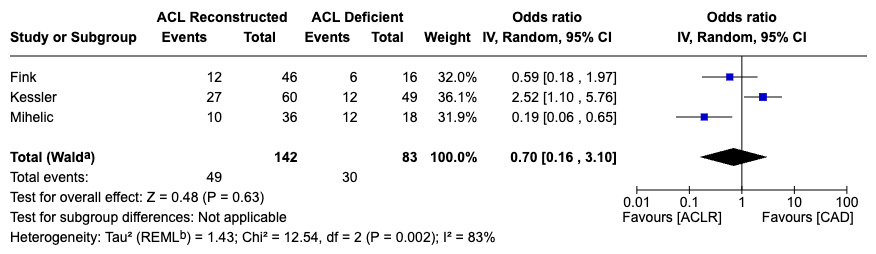


Appendix Figure 3. Meta-analysis of low-impact athletes (Tegner < 7)

**TABLES:**

**Appendix Table A:** Search strategy

| Strategy | Studies |
| --- | --- |
| (anterior cruciate ligament or anterior cruciate deficien* or anterior cruciate ligament deficien* or chronic anterior cruciate ligament deficien* or ACL rupture or ACL deficien* or ACL tear or chronic ACL injury or chronic ACL).mp. [mp=title, book title, abstract, original title, name of substance word, subject heading word, floating sub-heading word, keyword heading word, organism supplementary concept word, protocol supplementary concept word, rare disease supplementary concept word, unique identifier, synonyms, population supplementary concept word, anatomy supplementary concept word] | 29898 |
| (radiograph* or X-ray* or CT or computed tomography).mp. [mp=title, book title, abstract, original title, name of substance word, subject heading word, floating sub-heading word, keyword heading word, organism supplementary concept word, protocol supplementary concept word, rare disease supplementary concept word, unique identifier, synonyms, population supplementary concept word, anatomy supplementary concept word] | 1857473 |
| (tibial slope or tibial translation or radiographic parameters or arthritis or instability or rotation or anterior translation or posterior translation or osteophyte or Joint space narrowing or Subchondral or Bone marrow lesions or condyle or fracture or degeneration or deformity).mp. [mp=title, book title, abstract, original title, name of substance word, subject heading word, floating sub-heading word, keyword heading word, organism supplementary concept word, protocol supplementary concept word, rare disease supplementary concept word, unique identifier, synonyms, population supplementary concept word, anatomy supplementary concept word] | 1075199 |
| 1 and 2 and 3 | 2187 |

**Appendix Table B – Study Characteristics**

| **Title** | **Author** | **Year** | **Sample Size (Total)** | **Mean Age (Total)** | **Sample Size (ACLD)** | **Mean Age (ACLD)** | **Sample Size (Control)** | **Control Group Criteria** | **Baseline Equivalence of Groups (Y/N)** | **Meniscal Injuries/ Surgery (n, %)** | **Sex [Total n, Male (%), ACLD Male (%)]** | **Time Since Injury (range)** | **Pre-Injury Activity Level** |
| --- | --- | --- | --- | --- | --- | --- | --- | --- | --- | --- | --- | --- | --- |
| Prospective Comparative Studies | | | | | | | | | | | | | |
| Long-term Outcome of Operative or Nonoperative Treatment of Anterior Cruciate Ligament Rupture -Is Sports Activity a Determining Variable? | Fink | 2001 | 71 | 33.2 | 25 | 32.3 (9.9) | 46 | ACL reconstruction | Y (age, activity, injuries) | NR | 71, 55(77.5%), 18/25 (72%) | 132.2 + 8.1 months | IKRS: 1.78 (1 = Weekend, 2 = Recreational) |
| Treatment for acute anterior cruciate ligament tear: five year outcome of randomised trial | Frobell | 2013 | 121 | 26 | 29 | 26.4 (4.91) | Early ACLR: 59 Delayed ACLR: 30 | ACL reconstruction | Y (all) | Surgery: ACLD: 27/29; ACLR: 79/89 | 89, 66/89 (74.2%), 20/29 (69.0%) | 5 years | Tegner: 9 (7-9) |
| Function, osteoarthritis and activity after ACL-rupture: 11 years follow-up results of conservative versus reconstructive treatment | Kessler | 2008 | 109 | 30.7 years 12.5-54.0) | 49 | NR | 60 | ACL reconstruction | Y (all) | Surgery: ACLD: 18/68 ACLR: 7/68 | 109, 68 (62.4%), NR | 11.1 years (7.5-16.3) | Tegner: 5.9 (2-10) |
| Ten year follow-up study comparing conservative versus operative treatment of anterior cruciate ligament ruptures. A matched-pair analysis of high level athletes | Meuffels | 2008 | 50 | 37.7 | 25 | 37.8 (6.8) | 25 | ACL reconstruction | Y (all) | Injury: No difference (p>.05), Surgery: ACLD: 10/25, ACLR: 3/25 | 50, 38 (76%), 19 (76%) | >10 years | Tegner: 9 (6-10) |
| Long-term results of anterior cruciate ligament reconstruction: a comparison with non-operative treatment with a follow-up of 17-20 years | Mihelic | 2011 | 54 | 25.4 | 18 | 25.5 | 36 | ACL reconstruction | Y (age, activity) | NR | 54, 44 (81.5%), NR | 17-20 year follow up | Tegner: 5 (2-9) |
| Clinical, Functional, and Radiologic Outcome in Team Handball Players 6 to 11 Years after Anterior Cruciate Ligament Injury: A Follow-up Study | Myklebust | 2003 | 79 | NR | 22 | NR | 57 | ACL reconstruction | Y | NR | 79, 29 (36.7%), 9 (40.9%) | 7.8 years (6-11) | **Competitive handball players** |
| Prevalence of Tibiofemoral Osteoarthritis 15 Years After Nonoperative Treatment of Anterior Cruciate Ligament Injury: A Prospective Cohort Study | Neuman | 2008 | 79 | 26 (SD 8, range 15-43) | 79 | 26 (SD 8, range 15-43) | 79 | ACL reconstruction | Y (activity) | NR | 79, NR, NR | 15.7 years (SD 1.4) | Tegner: 7 (3-9) |
| Low Rates of Radiographic Knee Osteoarthritis 5 Years After ACL Reconstruction or Rehabilitation Alone: The Delaware-Oslo ACL Cohort Study | Pedersen | 2021 | 187 | Early ACLR group (n=167): 24.7 ± 8.7 years  Delayed ACLR group (n=30): 24.4 ± 9.4 years  Progressive Rehabilitation group (n=65): 31.9 ± 10.9 years | 65 | 31.9 ± 10.9 | 65 | ACL reconstruction | N (age, activity diff) | NR | 276, 155 (56%), 29 (45%) | 5 years | Athletes playing sport >2x/week |
| Reconstructive versus non-reconstructive treatment of anterior cruciate ligament insufficiency. A retrospective matched-pair long-term follow-up | Streich | 2011 | 80 | 25.8 (17-39) | 80 | 24.0 ± 6.5 (range 17-38) | 40 | ACL reconstruction | Y (all) | Surgery: ACLD: 16/40, ACLR: 4/40 | 80, 56 (70%), 28 (70%) | 15.2 ± 0.69 years (14–16) | Tegner: 7.1 (6-9) |
| No difference in osteoarthritis after surgical and non‐surgical treatment of ACL‐injured knees after 10 years | Tsoukas | 2016 | 32 | 31.94 | 15 | Median: 33 (range 25-39) | 17 | ACL reconstruction | Y (age) | Surgery: ACLD: 0/15, ACLR: 0/17 | 32, 32 (100%), 15 (100%) | 10.3 years (10-11) | Tegner: 7 (4-7) |
| Twenty-Year Follow-up Study Comparing Operative Versus Nonoperative Treatment of Anterior Cruciate Ligament Ruptures in High-Level Athletes | Van Yperen | 2018 | 50 | 37.6 ± 6.1 operative group, 37.8 ± 6.8 non-operative group | 25 | 37.8 ± 6.8 | 25 | ACL reconstruction | Y | Surgery: ACLD: 10/25 ACLR: 4/25 | 50, 38 (76%), 19 (76%) | Median 24.1 years (IQR 22.6-27.0) | Tegner: 9 (7-9) |
| Prospective Cohort Studies | | | | | | | | | | | | | |
| Radiological sign of chronic anterior cruciate ligament deficiency | Fairclough | 1990 | 78 | NR | 38 | NR | 40 | Randomly selected patients with intact ACL confirmed by arthroscopy, similar age range to ACLd group | N | NR | NR | >6 mo | NR |
| Non-operative treatment of ACL injury is associated with opposing subjective and objective outcomes over 20 years of follow-up | Gfoller | 2019 | 21 | 53.1±9.2 | 21 | 53.1±9.2 | N/A | N/A | NA | NR | 21, 71.4% (15) | 20.7 years | IKRS: 1.8 (SD: 1.0) |
| Posttraumatic anterior cruciate ligament insufficiency as a cause of osteoarthritis in a knee joint | Kannus | 1989 | 77 | 30 ± 11 | 40 | 30 + 11 | 40 | Intact contralateral knees | NA | NR | 77, 75.3% (58) | 8.0 + 2.1 years | NR |
| Long-term outcome of anterior cruciate ligament tear without reconstruction: a longitudinal prospective study | Konrads | 2016 | 127 | 33.8 (18-66) - at time of injury | 50 | 33.8 (range 18-66) | N/A | N/A | NA | Surgery: 5/99 | 236, 178 (78%) | 27.1 years (21.3-31.5) | NR |
| The Untreated Anterior Cruciate Ligament Rupture | McDaniel | 1982 | 49 | 33 (range 16-70) | 49 | 33 (range 16-70) | NA | NA | NA | NR | 49, 41 (83.7%) | 14 years (range: 8-47 years) | NR |
| Four to ten year followup of unreconstructed anterior cruciate ligament tears | Pattee | 1989 | 40 | 27 (16-46) | 40 | 28 | N/A | N/A | NA | Surgery: 2/40 | 49, 37 (75.5%), 12 (24.5%) | 67 months (48 to 122 months) | NR |
| A clinical and radiographic analysis of 127 anterior cruciate deficient knees | Sherman | 1988 | 127 | 28.6 | 47 patients (37%) had injury duration >5 years  28 NApatients had interval times >10 years  12 patients had interval times >20 years | 49.5 (range 30-63) | N/A | N/A | NA | NR | 127, 94 (74%) | 6.6 years | NR |
| The natural history of untreated anterior cruciate ligament tears in recreational athletes | Shirakura | 1995 | 56 | 28.9 ± 10.3 (14-50) | 56 | 28.9 (14-50) | N/A | N/A | NA | Surgery: 4/56 | 56, 24 (43%) | 5.5 years ± 27.1 months | NR; “Recreational athletes” |

NR, Not reported; ACLD, ACL-deficient; N/A, not applicable; IKRS, Innsbruck Knee Rating Scale (1 = Weekend, 2 = Recreational 3-5/wk)

**Appendix Table C: Surgical and meniscal injury details**

| **Title** | **Author** | **Year** | **Sample Size (ACLD)** | **Sample Size (Control)** | **Time to Follow-Up** | **ACLR Graft** | **Femoral Tunnel Technique** | **Other Procedure Details** | **Meniscectomy** | **Meniscal Repair** | **Concurrent Ligament Injuries** |
| --- | --- | --- | --- | --- | --- | --- | --- | --- | --- | --- | --- |
| Long-term Outcome of Operative or Nonoperative Treatment of Anterior Cruciate Ligament Rupture -Is Sports Activity a Determining Variable? | Fink | 2001 | 25 | 46 | 132.2 + 8.1 months | BPTB | NR | Open technique with medial parapatellar arthrotomy. The graft was fixed with sutures tied over a bone bridge. | Control: n=12 (n= 4 post-injury, n=6 medial tears, n=5 lateral tears);  ACLR: n=23 (n=7 post-injury, n=11 medial tears, n=12 lateral tears) | NR | Control: n=4 medial collateral ligament sprains; ACLR: n=7 medial collateral ligament sprains (all grade 1+ additional medial laxity) |
| Treatment for acute anterior cruciate ligament tear: five year outcome of randomised trial | Frobell | 2013 | 29 | 89 | 5 years | BPTB (n=40); Hamstring (n=51) | NR | Single bundle reconstruction | Control: n=32;  ACLR: n=29 | NR | NR |
| Function, osteoarthritis and activity after ACL-rupture: 11 years follow-up results of conservative versus reconstructive treatment | Kessler | 2008 | 49 | 60 | 11.1 years (7.5-16.3) | BPTB | NR | Extra-articular screw fixation of tibia and femur in arthroscope-assisted and mini-arthrotomy technique | Meniscal injuries excluded at baseline | NR | NR |
| Ten year follow-up study comparing conservative versus operative treatment of anterior cruciate ligament ruptures. A matched-pair analysis of high level athletes | Meuffels | 2008 | 25 | 25 | >10 years | BPTB | NR | Non-resorbable interference screw fixation | Control: n=20,  ACLR: n=17 | NR | NR |
| Long-term results of anterior cruciate ligament reconstruction: a comparison with non-operative treatment with a follow-up of 17-20 years | Mihelic | 2011 | 18 | 36 | 17-20 year follow up | BPTB | NR | Open reconstruction with two skin incisions, graft fixed with non-absorbable sutures over cortical screws, 10-15 full knee flexions performed before fixing tibial graft end | Control: n=11 (n=6 medial, n=5 combined);  ACLR: n=23 (n=10 medial, n=13 combined) | NR | NR |
| Clinical, Functional, and Radiologic Outcome in Team Handball Players 6 to 11 Years after Anterior Cruciate Ligament Injury: A Follow-up Study | Myklebust | 2003 | 22 | 57 | 7.8 years (6-11) | BPTB | NR | NR | NR | NR | NR |
| Prevalence of Tibiofemoral Osteoarthritis 15 Years After Nonoperative Treatment of Anterior Cruciate Ligament Injury: A Prospective Cohort Study | Neuman | 2008 | 79 | 79 | 15.7 years (SD 1.4) | BPTB | NR | NR | Control: n=46; 21/46 post-injury | NR | Medial collateral ligament: n=22 |
| Low Rates of Radiographic Knee Osteoarthritis 5 Years After ACL Reconstruction or Rehabilitation Alone: The Delaware-Oslo ACL Cohort Study | Pedersen | 2021 | 65 | 65 | 5 years | BPTB, Hamstring, Allograft | NR | NR | Control: n=0;  Early ACLR: n=18,  Delayed ACLR: n=3 | Early ACLR: n=45 medial, n=34 lateral Delayed ACLR: n=8 medial, n=7 lateral Control: n=7 medial, n=6 lateral | MCL (control: n=11, ACLR: n=45),  LCL (control: n=4, ACLR: n=4) |
| Reconstructive versus non-reconstructive treatment of anterior cruciate ligament insufficiency. A retrospective matched-pair long-term follow-up | Streich | 2011 | 80 | 40 | 15.2 ± 0.69 years (14–16) | BPTB | Transtibial | Metal interference screw fixation | Control: n=10;  ACLR: n=9 | NR | NR |
| No difference in osteoarthritis after surgical and non‐surgical treatment of ACL‐injured knees after 10 years | Tsoukas | 2016 | 15 | 17 | 10.3 years (10-11) | Hamstring | Anteromedial portal | Endobutton femoral fixation | NR | NR | NR |
| Twenty-Year Follow-up Study Comparing Operative Versus Nonoperative Treatment of Anterior Cruciate Ligament Ruptures in High-Level Athletes | Van Yperen | 2018 | 25 | 25 | Median 24.1 years (IQR 22.6-27.0) | BPTB | Transtibial | Interference screw fixation | Control: n=19 (n=10 post-injury period); n=13 medial, n=3 lateral, n=4 combined,  ACLR: n=18 (n=4 post-injury period); n=15 medial, n=6 lateral, n=3 combined | NR | NR |
| Radiological sign of chronic anterior cruciate ligament deficiency | Fairclough | 1990 | 38 | 40 | >6 mo | N/A | N/A | N/A | NR | NR | NR |
| Non-operative treatment of ACL injury is associated with opposing subjective and objective outcomes over 20 years of follow-up | Gfoller | 2019 | 21 | N/A | 20.7 years | N/A | N/A | N/A | n=7 total meniscal injuries at baseline (n=4 medial, n=1 lateral, n=2 bilateral) | NR | NR |
| Posttraumatic anterior cruciate ligament insufficiency as a cause of osteoarthritis in a knee joint | Kannus | 1989 | 40 | 40 | 8.0 + 2.1 years | N/A | N/A | N/A | n=7 medial,  n=1 lateral,  n=1 bilateral;  n=9 post-injury (n=8 medial, n=1 lateral) | NR | NR |
| Long-term outcome of anterior cruciate ligament tear without reconstruction: a longitudinal prospective study | Konrads | 2016 | 181 | N/A | 27.1 years (21.3-31.5) | N/A | N/A | N/A | n=67 medial, n=48 lateral | n=1 (outside-in reconstruction) | NR |
| The Untreated Anterior Cruciate Ligament Rupture | McDaniel | 1982 | 49 | NA | 14 years (range: 8-47 years) | N/A | N/A | Initial arthrotomy: no ACL treatment in n=18, excision of loose tags in n=32, direct repair with one suture in n=3; later reconstruction: pes anserinus transfer in n=3, Ellison-type reconstruction in n=1 | n=30 medial meniscectomies,  n=8 lateral meniscectomies,  n=7 both menisci removed | NR | NR |
| Four to ten year followup of unreconstructed anterior cruciate ligament tears | Pattee | 1989 | 40 | N/A | 67 months (48 to 122 months) | N/A | N/A | N/A | n=25 medial meniscus tears (51%),  n=21 lateral meniscus tears (43%),  n=13 both menisci torn (27%) | NR | NR |
| A clinical and radiographic analysis of 127 anterior cruciate deficient knees | Sherman | 1988 | 87 | N/A | 6.6 years | N/A | N/A | N/A | Control: n=39 | NR | MCL (n=67) |
| The natural history of untreated anterior cruciate ligament tears in recreational athletes | Shirakura | 1995 | 56 | N/A | 5.5 years ± 27.1 months | N/A | N/A | N/A | Control: n=56 | NR | MCL (n=20) |

NR, Not reported; ACLD, ACL-deficient; N/A, not applicable

**Appendix Table D: Key radiographic findings from each study**

| **Title** | **Author** | **Year** | **Radiographic Outcomes** | **Relevant Radiographic Findings** |
| --- | --- | --- | --- | --- |
| Prospective Comparative Studies | | | | |
| Long-term Outcome of Operative or Nonoperative Treatment of Anterior Cruciate Ligament Rupture -Is Sports Activity a Determining Variable? | Fink | 2001 | Fairbank classification (OA) | Both groups developed similar mild-to-moderate degenerative changes based on modified Fairbank classification.  Baseline: ACLD (n=25): 16 (65.2%) grade 0, 9 (34.8%) grade 1.  FU I (74.2 + 8.1 months): ACLD (n=25): 10 (39.1%) grade 0, 8 (30.4%) grade 1, 4 (17.4%) grade 2, 3 (13%) grade 3. **13 patients developed moderate-severe arthritis, 7 patients developed severe arthritis.** FU II (132.2 + 8.1 months): ACLD (n=25): 4 (17.4%) grade 0, 9 (34.8%) grade 1, 3 (13%) grade 2, 9 (34.8%) grade 3. **12 patients developed moderate-severe arthritis, 12 patients developed severe arthritis.**  There was statistically significant difference between both follow-up timepoints (p<0.05). Both operative and non-operative groups showed similar progression of degeneration over time. At FU1 for the non-operative group, there were **significant correlations (p<0.05) between arthritic change and the level of participation in pivoting sports (r=0.64).** Furthermore, **there was a significant correlation (p<0.05) between degree of arthrosis and the amount of meniscus resection performed at time of initial arthroscopy (medial: r=0.60, lateral: r=0.71) in the ACLD group, but not the control (ACLR) group.** |
| Treatment for acute anterior cruciate ligament tear: five year outcome of randomised trial | Frobell | 2013 | Osteoarthritis Research International atlas | **5/26 patients in the ACLD group had radiographic osteoarthritis (medial OA: n=2, lateral OA: n=1, patellofemoral OA: n=2**). 23/58 patients in the early ACLR group had radiographic OA (medial OA: n=6, lateral OA: n=5, patellofemoral OA: n=14), and 7/29 in the delayed ACLR had OA (medial OA: n=1, patellofemoral OA: n=6). No difference between groups (p>0.05). |
| Function, osteoarthritis and activity after ACL-rupture: 11 years follow-up results of conservative versus reconstructive treatment | Kessler | 2008 | Kellgren and Lawrence grading for OA assessment | At 11.1 years (7.5 - 16.3 years) followup, in the Conservative Treatment group: 61% (30/49) had (Grade 0), 14% (7/49) had (Grade I), **20% (10/49) had (Grade II), and 4% (2/49) had (Grade III).** Overall risk (grade >2) is 24% in conservative group.  Odds ratio of a higher OA rate, adjusted for gender, Tegner score, and time to FU: ACLR surgery: 2.8 (1.06-7.5). Greater age (>10 yrs): 1.7 (1.04-2.9), higher BMI: 1.2 (1.02-1.3) also predicted arthritis. |
| Ten year follow-up study comparing conservative versus operative treatment of anterior cruciate ligament ruptures. A matched-pair analysis of high level athletes | Meuffels | 2008 | Kellgren & Lawrence grading for OA assessment | ACLD group: Grade 0: 8 patients (32%), Grade 1: 10 patients (40%), **Grade 2: 4 patients (16%), Grade 3: 3 patients (12%), Grade 4: 0 patients (0%). 28% (7 patients) had knee radiographic OA (score ≥2).** Contralateral (unaffected) knees: Grade 0: 37 knees (74%), Grade 1: 11 knees (22%), Grade 2: 2 knees (4%), Grade 3&4: 0 knees (0%). Only 4% showed radiographic OA.  No statistically significant difference in OA between operative and conservative groups (p=0.145). Trend toward higher OA in operative group (48% vs 28%). Contralateral knees showed very low OA rates (4%). Most severe cases (Grade 3) equally distributed between groups (12% each). No Grade 4 OA in either group. |
| Long-term results of anterior cruciate ligament reconstruction: a comparison with non-operative treatment with a follow-up of 17-20 years | Mihelic | 2011 | IKDC radiographic grading scale | At 17-20 years post-injury, the ACLD Group (n=18): **100% of knees had IKDC > B, 56% showed severe osteoarthritic changes (IKDC > C).** Higher prevalence of severe changes compared to reconstructed group (statistically significant, P<0.05). Highest percentage of severe changes seen in patients with combined meniscal injury (IKDC B: 1/18, D: 8/18 in ACLD + meniscus injury group, versus IKDC B: 4/18, D: 4/18 in ACLD only group). |
| Clinical, Functional, and Radiologic Outcome in Team Handball Players 6 to 11 Years after Anterior Cruciate Ligament Injury: A Follow-up Study | Myklebust | 2003 | Radiological diagnosis of gonarthrosis | Nonoperative group (n=13): **6 subjects (46%) developed gonarthrosis**. No statistically significant correlation between radiologic findings and IKDC pain score (p=0.27) or radiologic findings and Lysholm pain scores (p=0.79) |
| Prevalence of Tibiofemoral Osteoarthritis 15 Years After Nonoperative Treatment of Anterior Cruciate Ligament Injury: A Prospective Cohort Study | Neuman | 2008 | Radiographic OA | At mean 15.7 (1.4) years followup, **7/62 non-reconstructed knees developed OA (compared to 6/17 in ACLR group, p = .03).** Meniscectomy Impact: All 13 patients with OA had undergone meniscectomy. 0 of 44 non-meniscectomized knees developed OA (p < .0001). OA developed in same compartment as meniscal tear. There was no difference in TF OA between patients with medial collateral ligament injuries (grades 1-3) or no medial collateral ligament injury. |
| Low Rates of Radiographic Knee Osteoarthritis 5 Years After ACL Reconstruction or Rehabilitation Alone: The Delaware-Oslo ACL Cohort Study | Pedersen | 2021 | Kellgren and Lawrence radiological scoring system, mimimum joint space width (mJSW) difference to contralateral knee | ACLD Group (n=54**): K&L grade ≥2: 2% (1/54), K&L grade 2/osteophyte: 19% (10/54),** K&L grade ≥1: 32% (17/54).  Other injuries (eg: meniscus, MCL, LCL; n=15): K&L grade ≥2: 0% (0/15), K&L grade 2/osteophyte: 20% (3/15), K&L grade ≥1: 33% (5/15) vs No other injuries (n=39): K&L grade ≥2: 3% (1/39), K&L grade 2/osteophyte: 18% (7/39), K&L grade ≥1: 4%% (2/47). Contralateral knee K&L grade ≥2: 6% (3/54). Medial mJSW difference: 0.0 ± 0.7 mm. Lateral mJSW difference: -0.0 ± 0.9 mm. No statistically significant differences between groups for any radiographic outcome (p > 0.05). |
| Reconstructive versus non-reconstructive treatment of anterior cruciate ligament insufficiency. A retrospective matched-pair long-term follow-up | Streich | 2011 | IKDC radiographic grading scale | At 15.2 ± 0.69 (14–16) post-injury, the ACLD Group (n=40): 22 knees (55%) progressed in radiological OA grade over follow-up; at baseline, IKDC Grade A: 38 patients, Grade B: 2 patients; at follow-up IKDC Grade A: 18 patients, Grade B: 7 patients, **Grade C: 4 patients, Grade D: 11 patients**. No correlation found between radiographic OA and KT-1000 measurements, Age, BMI, Gender, Time from injury to treatment, Initial/secondary meniscal surgery, Functional outcome scores. Significant correlation between positive pivot shift test and radiographic OA grading (p<0.001). This correlation was independent of treatment group |
| No difference in osteoarthritis after surgical and non‐surgical treatment of ACL‐injured knees after 10 years | Tsoukas | 2016 | IKDC radiographic grading scale | At 10.3 years (range 10–11), the ACLD Group (n=15**): 5 patients (33.3%) showed grade C or D IKDC changes**.  No correlation in ACLD group between OA and age (p>.05) or between groups. |
| Twenty-Year Follow-up Study Comparing Operative Versus Nonoperative Treatment of Anterior Cruciate Ligament Ruptures in High-Level Athletes | Van Yperen | 2018 | Kellgren and Lawrence radiological scoring system | At 12.0 (11.0-13.0) years, 12/25 patients from the operative group, and **7/25 from the nonoperative group had OA** (KL >2), and 3/25 in each group had KL >3. At 24.1 (22.6-27.0) years, 20/25 patients (80%) from the operative group had knee OA (KL >2) compared **with 17/25 patients (68%) from the nonoperative group**, and 3/25 in the ACLR group versus 5/25 in the ACLD group had KL >3. 2/25 contralateral knees had OA. There were no statistically significant difference regarding knee OA between groups (P = .508). **Of the 19 ACLD patients who underwent meniscectomy, 13/19 had knee OA at 20-year followup (compared to 2/6 in the non-meniscectomy group).** |
| Prospective Cohort Studies | | | | |
| Radiological sign of chronic anterior cruciate ligament deficiency | Fairclough | 1990 | Presence of osteophytes, presence of degenerative changes | At 6 months – 8 years post-injury, 36/38 had osteophyte on medial side of intercondylar notch, 28/38 had an osteophyte on the medial tibial spine. **16/38 had degeneration on XR, compared to 12/40 in the control group (ACL intact).** |
| Non-operative treatment of ACL injury is associated with opposing subjective and objective outcomes over 20 years of follow-up | Gfoller | 2019 | Fairbank scores | 20.7 years (248.3 ± 8.6 months) after injury, Fairbank scores increasing from 0.4 ± 0.5 at baseline to 2.2 ± 1.2. Arthritis was more severe in the injured versus contralateral knee (1.1±0.7; Z=−3.132, p=0.002, r=0.54). **58.8% of the patients showed extensive arthritic changes, 17.6% intermediate**, 5.9% low and 17.6% no radiographic changes after 20 years. **Notably, the extent of lateral meniscus removal during initial treatment strongly correlated with the development of lateral compartment arthritis (R = 0.550, p = 0.022).** Significant differences in the degree of arthritis between subgroups of subjects with no or only minor compared to larger extent resection of the lateral meniscus were found (Z = − 2.092, p = 0.036, r = 0.51). The degree of sports participation did not correlate with overall arthritic progression. The study also found no significant relationship between medial meniscal resection and medial compartment arthritis. |
| Posttraumatic anterior cruciate ligament insufficiency as a cause of osteoarthritis in a knee joint | Kannus | 1989 | Radiographic arthritis knee scoring scale | At 7.8 ± 2.0 years follow-up study, chronic ACL insufficiency (n=77) led to characteristic patterns of osteoarthritic changes that varied based on injury severity. In partial ACL tears (n=37), about 83% of changes concentrated in specific areas: osteophytes and subchondral sclerosis in the medial femoral condyle (15 patients), medial tibial condyle (16 patients), tibial eminence (27 patients), and patella (16 patients), along with joint space narrowing medially (7 patients) and laterally (5 patients). Complete ACL tears (n=40) showed similar patterns but with significantly more severe changes, affecting more patients: medial femoral (27 patients) and tibial (24 patients) condyles, tibial eminence (28 patients), and patella (31 patients), with increased joint space narrowing both medially (16 patients) and laterally (19 patients). 9/40 had meniscal injury treated with open meniscectomy. **70% (28/40) of complete ACLD patients developed clear posttraumatic osteoarthritis compared to only 14% (5/37) in partial tears**. The radiological scores averaged 96±8 (good) for partial tears versus 83±11 (poor) for complete tears (p<0.001), demonstrating that the severity of initial ACL injury significantly influenced the development of radiographic osteoarthritic changes. Meniscectomized knee scores were lower than those with intact menisci (80 + 14 versus 84 + 11). |
| Long-term outcome of anterior cruciate ligament tear without reconstruction: a longitudinal prospective study | Konrads | 2016 | Sherman P-score, D-score, and T-score | At 27.1 (21.3-31.5) years post-op, Sherman scores (P-score, D-score, T-score) worsened between 12 and 27 year follow-up; Mean P-score decreased from 7.2 (±1.9) to 5.5 (±1.0) (p=0.03), Mean D-score decreased from 14.4 (±2.1) to 9.9 (±1.9) (p=0.02), Mean T-score decreased from 21.6 (±2.0) to 15.4 (±1.5) (p=0.02). **Patients with meniscal tears showed worse Sherman scores compared to those with intact menisci.** |
| The Untreated Anterior Cruciate Ligament Rupture | McDaniel | 1982 | Radiographic score (0 to 4) | **At 10 years, 11/53 knees had arthritic changes and 3/53 had severe changes. At 14 years, 17/45 knees had signs of arthritis, and 5/45 knees had severe arthritis changes. A relationship between varus knee, medial meniscectomy, and medial compartment OA was observed.** Of the 16 knees with medial joint space narrowing/OA, 13 were varus and had undergone medial meniscectomy. Other factors associated with arthritic changes were: higher BMI, lateral meniscectomy, higher age, restricted sports activity. PROMs (HSS score) and the degree of arthritis were inversely correlated. |
| Four to ten year followup of unreconstructed anterior cruciate ligament tears | Pattee | 1989 | Radiographic arthritic findings | At 5.6 years (4-10 years) post-injury, 7/20 patients (35%) had normal radiographs, 10 patients (50%) had mild femoral condyle flattening, and 3 patients (15%) had mild subchondral sclerosis and osteophyte formation. **0 patients (0%) showed joint space narrowing.** 13 patients (65%) showed mild degenerative changes, 7 patients (35%) had normal radiographs. **No obvious correlation to meniscal status.** |
| A clinical and radiographic analysis of 127 anterior cruciate deficient knees | Sherman | 1988 | Sherman score (OA assessment) | 79 months (6 months – 43 years) post-injury, radiographic scores were:  Group I: Isolated ACL (32 patients, 25%) - T-score: 24.5, D-score: 17.4, P-score: 7.1. Group II: ACL + Meniscectomy (15 patients, 12%) - T-score: 23.1, D-score: 16.9, P-score: 6.2. Group III: ACL + Meniscal Tear (13 patients, 10%) - T-score: 25.4, D-score: 17.4, P-score: 8.0. Group IV: ACL + Collateral Ligament (33 patients, 26%) - T-score: 24.8, D-score: 17.5, P-score: 7.3. Group V: ACL + Meniscectomy + Collateral Ligament (24 patients, 19%) - T-score: 21.4, D-score: 15.7, P-score: 5.7. Group VI: ACL + Meniscal Tear + Collateral Ligament (10 patients, 8%) - T-score: 22.0, D-score: 16.4, P-score: 5.6. Group V had the worst radiographic scores overall. Groups with meniscectomy (II and V) showed worse scores than those with just meniscal tears (III and VI). Groups III and VI (with retained meniscal tears) demonstrated less degeneration than groups with meniscectomy. Statistical significance was found between groups (F ratio <0.01 for T-score, D-score, and P-score). **In the 28 cases with interval >10 years, 18 (65%) had joint space narrowing, and degenerative score less than 16. Of these, 6/18 had prior meniscectomy. In the 12 cases with interval >20 years, 11 (83%) had joint space narrowing. Of these, 7/12 (58%) had prior meniscectomy; 4/5 without meniscectomy had arthritis. Coefficients between D-score and: interval time (-0.704), age (-0.536), medial meniscectomy (-0.223), and KDS (+0.222).** |
| The natural history of untreated anterior cruciate ligament tears in recreational athletes | Shirakura | 1995 | Radiographic Assessment of OA changes | Follow-up Radiographic Changes (n=46): Overall **15/46 patients (33%) showed radiographic differences between injured vs uninjured knee.** Specific changes observed: Squaring only: 3 cases, Squaring + subchondral sclerosis: 4 cases, Squaring + diffuse osteopenia: 1 case, Soft tissue ossification: 3 cases, Diffuse osteopenia: 2 cases, Combined soft tissue ossification + diffuse osteopenia: 2 cases. Osteoarthritic Changes: 8/46 patients (17%) showed Grade 1 osteoarthritis. Only 1 patient reported symptoms related to these changes. Clinical Relevance: Despite radiographic changes, most patients remained asymptomatic. Changes were generally mild in nature. Lower incidence of severe changes compared to previous studies |

ACLR, anterior cruciate ligament reconstruction; ACLD, ACL deficient; FU, follow-up

**Appendix Table E:** Radiographic scoring systems used to describe arthritic changes

| **SCORE NAME** | **DESCRIPTION** | **Definition of Osteoarthritis** | **Authors** |
| --- | --- | --- | --- |
| Kellgren/Lawrence radiological scoring system | Grade 0: absence of X-ray changes, Grade 1: Doubtful narrowing and possible osteophytes, Grade 2: definite osteophytes and possible joint space narrowing, Grade 3: moderate multiple osteophytes, definite joint space narrowing, sclerosis, bone deformity, Grade 4: large osteophytes, narrowing of joint space, severe sclerosis, definite bone deformity | K&L grade ≥2 (mild), ≥3 (moderate) | Kessler, Yperen, Neuman, Pederson |
| Sherman Score | Scoring system that combines both degenerative (0-18) and periarticular (0-10) changes in the knee. Total (T) score is 0-28, with 28 indicating a perfect joint. | Not defined in the paper, T-score <16 represents severe degeneration; <22 used as cutoff for OA | Konrads, Sherman |
| Fairbank Score | Grade 0: no changes, Grade 1: subchondral sclerosis, flattening of femoral condyles, Grade 2: extensive osteophyte formation, joint space narrowing <50%, Grade 3: joint space narrowing >50% | Fairbank score >1 (moderate), ≥2 (severe) | Fink, Gfoller |
| IKDC Evaluation Form (Radiographs) | Grade A: Normal, Grade B: Minimal joint space narrowing, Grade C: Moderate changes with joint space narrowing up to 50%, Grade D: Severe changes with axis deviation and subluxation | IKDC score >B (mild), ≥C (moderate-severe) | Mihelic, Tsoukas |
| Gonarthrosis Assessment | Defined as joint space narrowing with:  Loss of distance between tibia and femur in one compartment of half or more compared to other compartment of same knee, OR same compartment of other knee, OR less than 3mm joint space | Binary (Yes / No) | Myklebust |
| Osteoarthritis Research Society International (OARSI) atlas | 4-point scale (0-3) for:  Joint space narrowing (JSN) (0 = no JSN)  Osteophytes (0 = no changes) | JSN >2 or Osteophytes >2 in any compartment | Neuman, Frobell |

**Appendix Table F:** Statistical model fitting

| Moderate OA: |
| --- |
| linear: AIC = 0.1, SSE = 1.020, Equation: -0.009 + 0.037 * x  quadratic: AIC = 1.7, SSE = 1.002, Equation: -0.139 + 0.061 * x + -0.001 * x²  cubic: AIC = 3.6, SSE = 0.998, Equation: -0.269 + 0.100 * x + -0.004 * x² + 0.000 * x³  logarithmic: AIC = -0.5, SSE = 0.990, Equation: -0.549 + 0.415 * ln(x)  exponential: AIC = -57.8, SSE = 1.106, Equation: 0.171 * exp(0.070 * x)  Best model: exponential (AIC = -57.8) Equation: y = 0.171 * exp(0.070 * x) |
| Severe OA: |
| linear: AIC = -9.9, SSE = 0.393, Equation: -0.051 + 0.022 * x  quadratic: AIC = -9.4, SSE = 0.359, Equation: -0.256 + 0.060 * x + -0.001 * x²  cubic: AIC = -10.3, SSE = 0.298, Equation: 0.425 + -0.139 * x + 0.015 * x² + -0.000 * x³  logarithmic: AIC = -10.8, SSE = 0.372, Equation: -0.405 + 0.262 * ln(x)  exponential: AIC = -53.2, SSE = 0.448, Equation: 0.086 * exp(0.070 * x)  Best model: exponential (AIC = -53.2)  Equation: y = 0.086 * exp(0.070 * x) |
